# Supplementary material for: Evolution of giant pandoravirus revealed by CRISPR/Cas9
Source: Nat Commun. 2023 Jan 26;14:428. doi: 10.1038/s41467-023-36145-4 (PMC9879987; doi:10.1038/s41467-023-36145-4)

Supplementary Information File.

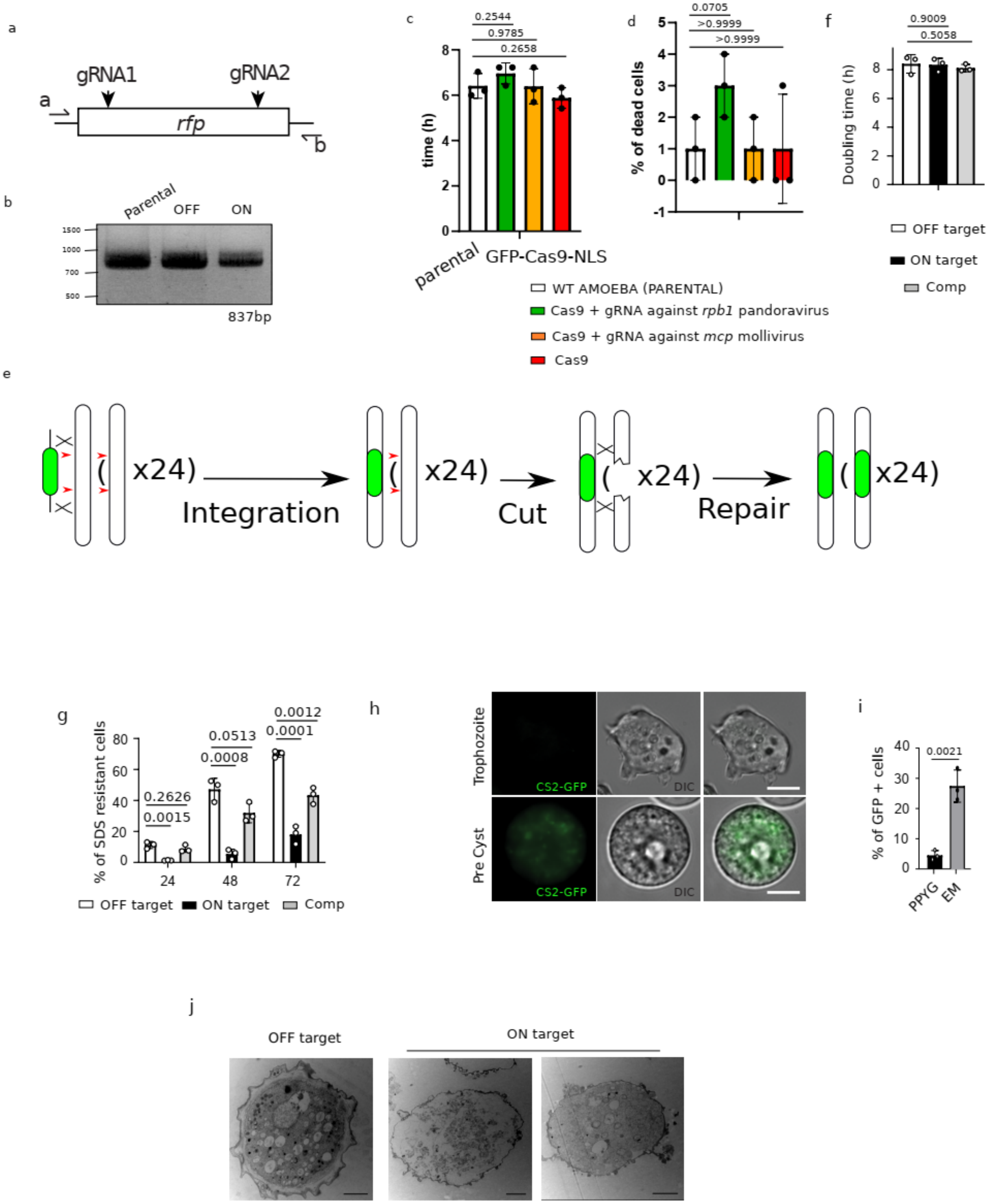

Figure S1. CRISPR/Cas9 allows manipulation of *A. castellanii*.

(a) Schematic representation of the rfp locus, guide targeting location and primer annealing sites.

(b) PCR of the genomic locus of the rfp shows no large deletions.

(c) Doubling time of the wild type amoeba (parental) and amoebas harboring GFP-Cas9-NLS was calculated. Presence or absence of gRNA targeting different viruses is shown. Data correspond to the mean ± SD of 3 independent experiments.

(d) Expression of CRISPR/Cas9 does not affect the percentage of death of *A. castellanii.* Presence or absence of gRNA targeting different viruses is shown. Data correspond to the mean ± SD of 3 independent experiments.

(e) Schematic representation of "mutagenic chain reaction" strategy[20]. Cas9 cleavage of the DNA at the site determined by the gRNA would allow insertion of the Cas9-gRNA cassette by homologous recombination. Continuous expression of Cas9-gRNA would allow continuous cleavage of unmodified alleles, which would propagate the Cas9-gRNA cassette in companion chromosomes if repair is performed by homologous recombination. Such strategy could facilitate the modification of the highly polyploid genome of *A. castellanii* (25n approximately)[21].

(f) Depletion of Cellulose synthase (CS) 1-3 does not affect the doubling time of *A. castellanii* trophozoytes. Comp: complemented strain. Data correspond to the mean ± SD of 3 independent experiments.

(g) Reduced resistance to SDS treatment was shown upon downregulation of CS. Resistance to SDS is associated to encystment and generation of a productive cyst wall. This data represents the mean and SD of 3 independent replicates. Comp: complemented strain.

(h) The CS1-GFP is express upon encystment. A representative image of trophozoite and pre-cyst is shown. Scale bar: 10μm.

(i) Quantification of representative pictures shown in (h). The mean ± SD of at least 200 amoebas (3 independent experiments (n=3)) is shown. PPYG: trophozoite media. EM: encystment media.

(j) Electron microscopy shows aberrant cyst formation upon downregulation of CS. Scale bar: 2μm. Micrographs are representative of 3 independent experiments.

Source data are provided as a Source Data file. Micrographs are representative of 3 independent experiments.

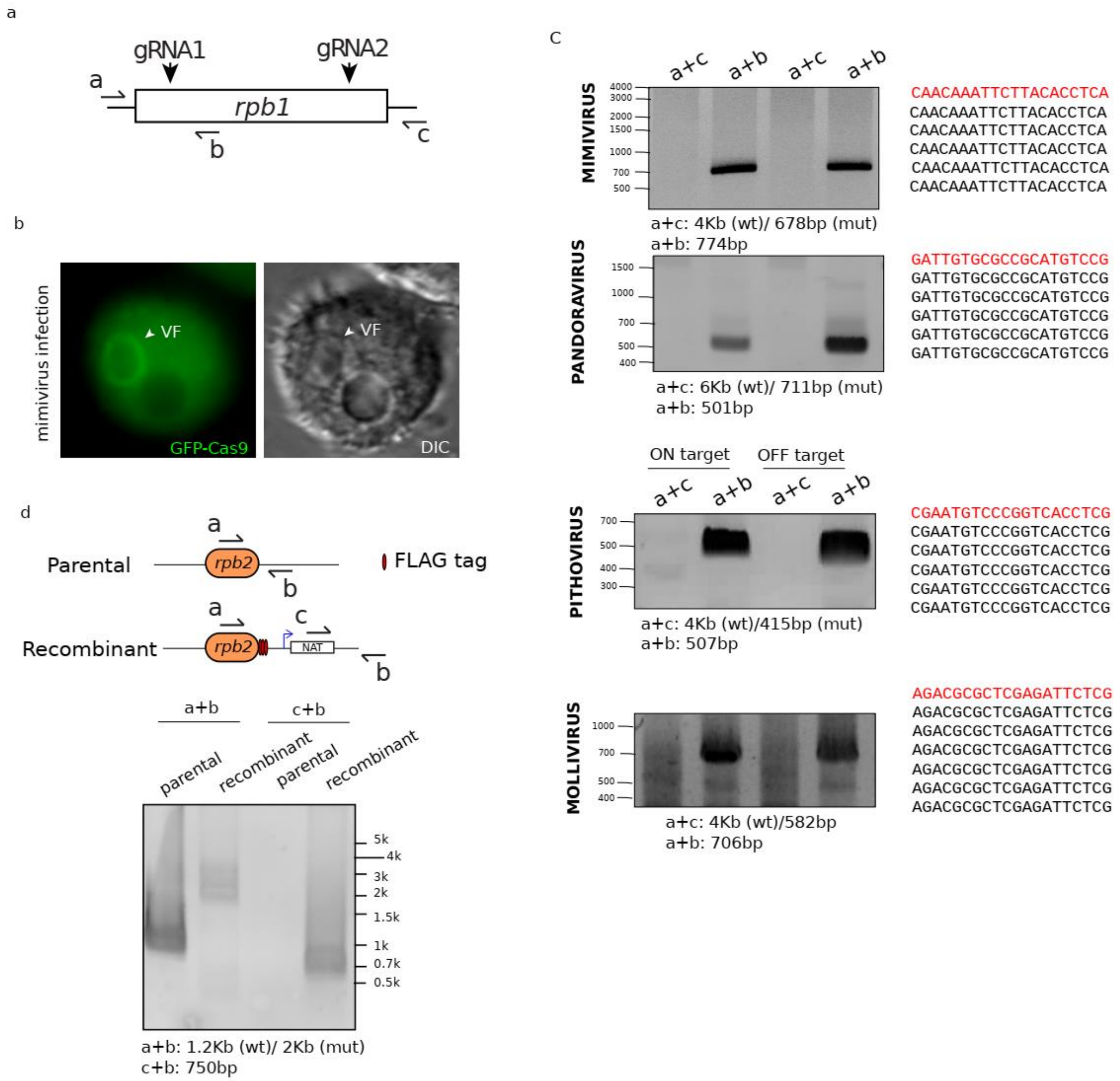

Figure S2. CRISPR/Cas9 allows manipulation of nuclear GVs.

(a) Schematic representation of the rpb1 locus, guide targeting location and primer annealing sites.

(b) Representative micrograph showing exclusion of GFP-Cas9 from the viral factory of mimivirus. VF: viral factory, white arrow head. An image of a non-infected cell is shown in Fig. 1a. Micrographs are representative of 3 independent experiments.

(c) PCR of the genomic locus of the rpb1 shows no noticeable deletions. Due to the length of the PCR product from primers a+c, when deletions are not present, wt PCR products are not detected with the current PCR setting. Sequencing results from a library from the PCR products demonstrate the lack of mutant viruses. The wild type sequence is shown in red. PCRs are representative of 3 independent experiments.

(d) PCR of the genomic locus of the *rpb2* demonstrate correct integration and clonality. A schematic representation of the wild-type and recombinant *rpb2* locus, and primer annealing sites are also shown. PCRs are representative of 2 independent experiments.

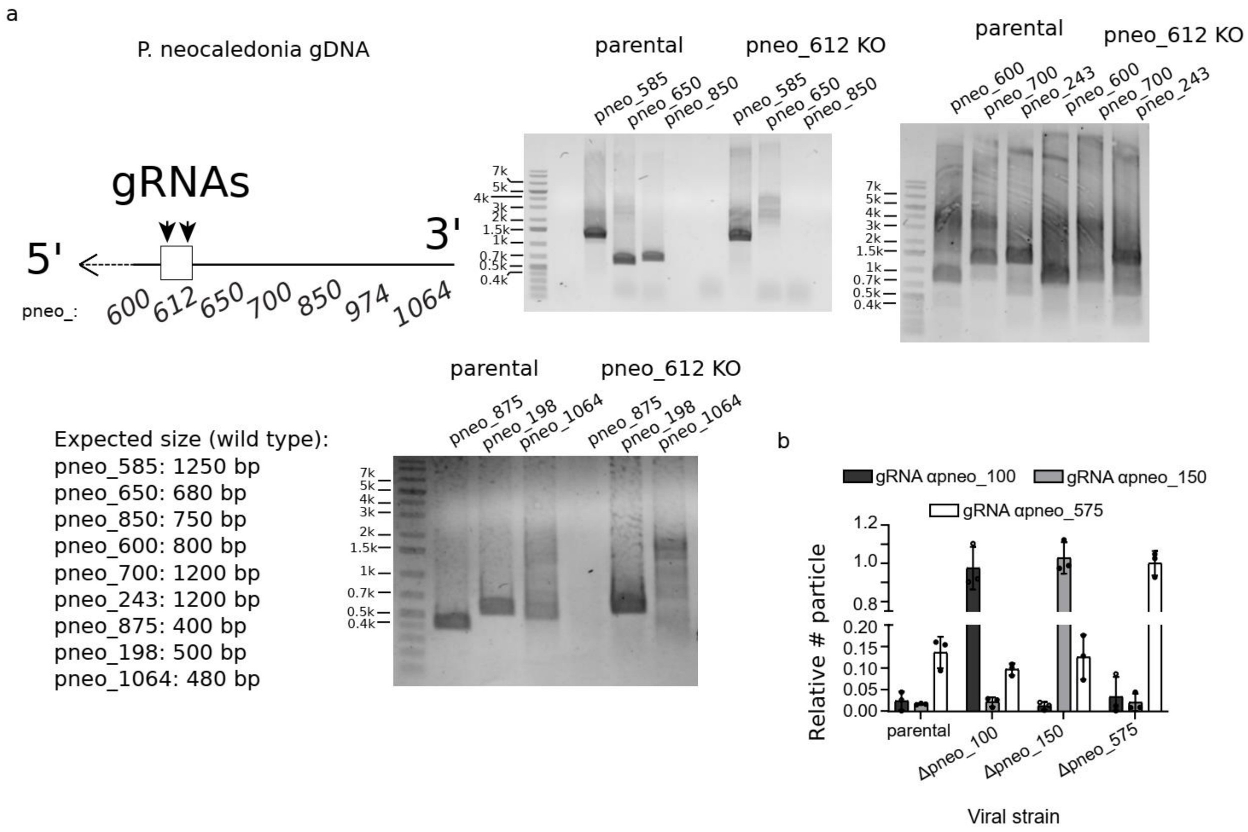

Figure S3. CRISPR/Cas9 targeting of pandoravirus genome demonstrates the presence of a core essential genome.

(a) PCR of different genomic locus shows large deletion at the 3' end of pandoravirus. Lack of specific PCR product indicates loss of the gene. Arrows indicate the gRNA targeting site.

(b) Relative quantification of the number of viral particles by parental (wild-type virus) or CRISPR/Cas9 modified viral strains after 24h of infection of CRISPR/Cas9 expressing

Acanthamoea cells. Modified viral strains were generated by continuous passage in CRISPR/Cas9 expressing cells until resistant viruses were obtained by each specific gRNA. Data is expressed relative to the growth of the strains in an off-target strain (gRNA agains the mcp of mollivirus). Data correspond to the mean ± SD of 3 independent experiments. α indicates the target of the gRNA.

Source data are provided as a Source Data file.

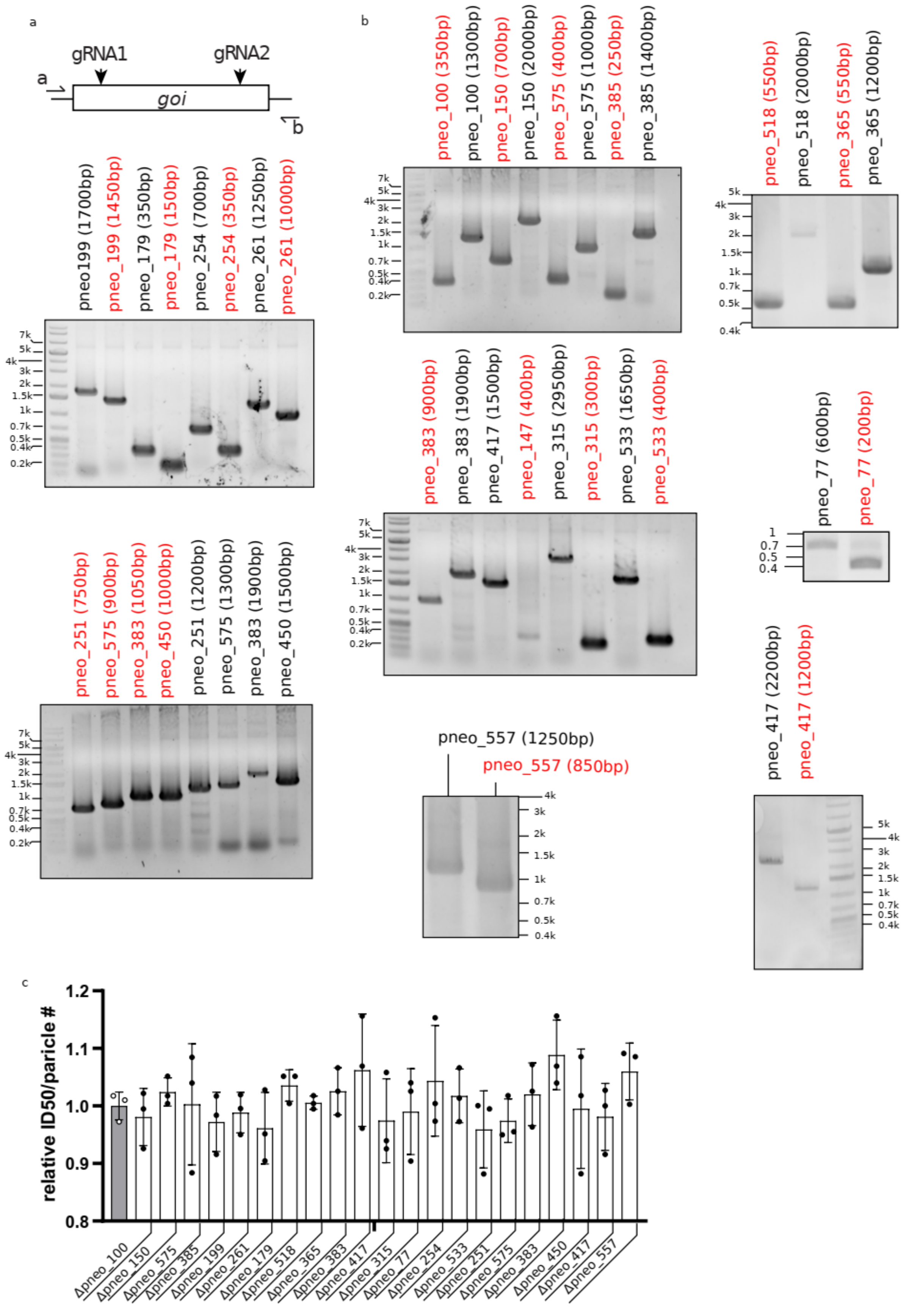
a
gRNA1
gRNA2
a
goi
b
pneo199 (1700bp)
pneo_199 (1450bp)
pneo_179 (350bp)
pneo_179 (150bp)
pneo_254 (700bp)
pneo_254 (350bp)
pneo_261 (1250bp)
pneo_261 (1000bp)
pneo_251 (750bp)
pneo_575 (900bp)
pneo_383 (1050bp)
pneo_450 (1000bp)
pneo_251 (1200bp)
pneo_575 (1300bp)
pneo_383 (1900bp)
pneo_450 (1500bp)
b
pneo_100 (350bp)
pneo_100 (1300bp)
pneo_150 (700bp)
pneo_150 (2000bp)
pneo_575 (400bp)
pneo_575 (1000bp)
pneo_385 (250bp)
pneo_385 (1400bp)
pneo_518 (550bp)
pneo_518 (2000bp)
pneo_365 (550bp)
pneo_365 (1200bp)
pneo_383 (900bp)
pneo_383 (1900bp)
pneo_417 (1500bp)
pneo_147 (400bp)
pneo_315 (2950bp)
pneo_315 (300bp)
pneo_533 (1650bp)
pneo_533 (400bp)
pneo_77 (600bp)
pneo_77 (200bp)
pneo_557 (1250bp)
pneo_557 (850bp)
pneo_417 (2200bp)
pneo_417 (1200bp)
7k
5k
4k
3k
2k
1.5k
1k
0.7k
0.5k
0.4k
0.2k
c
relative ID50/paricle #
1.2
1.1
1.0
0.9
0.8
Δpneo_100
Δpneo_150
Δpneo_575
Δpneo_385
Δpneo_199
Δpneo_261
Δpneo_179
Δpneo_518
Δpneo_365
Δpneo_383
Δpneo_417
Δpneo_315
Δpneo_77
Δpneo_254
Δpneo_533
Δpneo_251
Δpneo_575
Δpneo_383
Δpneo_450
Δpneo_417
Δpneo_557

Figure S4. Pandoravirus genome organization keeps traces of their smaller ancestors.

(a) Schematic representation of the *gene of interest* (*goi*) locus, guide targeting location and primer annealing sites.

(b) PCR of the genomic locus of the *goi*. Genotyping on parental viruses is shown in black while genotyping in recombinant viruses is marked in red. Expected PCR product size is indicated between parenthesis.

(c) Quantification of the infectious dose (ID50) per particle number was assessed as described in materials and methods. Values were normalized to wild type ID50/particle number (in grey). Data correspond to the mean ± SD of 3 independent experiments.

Source data are provided as a Source Data file.

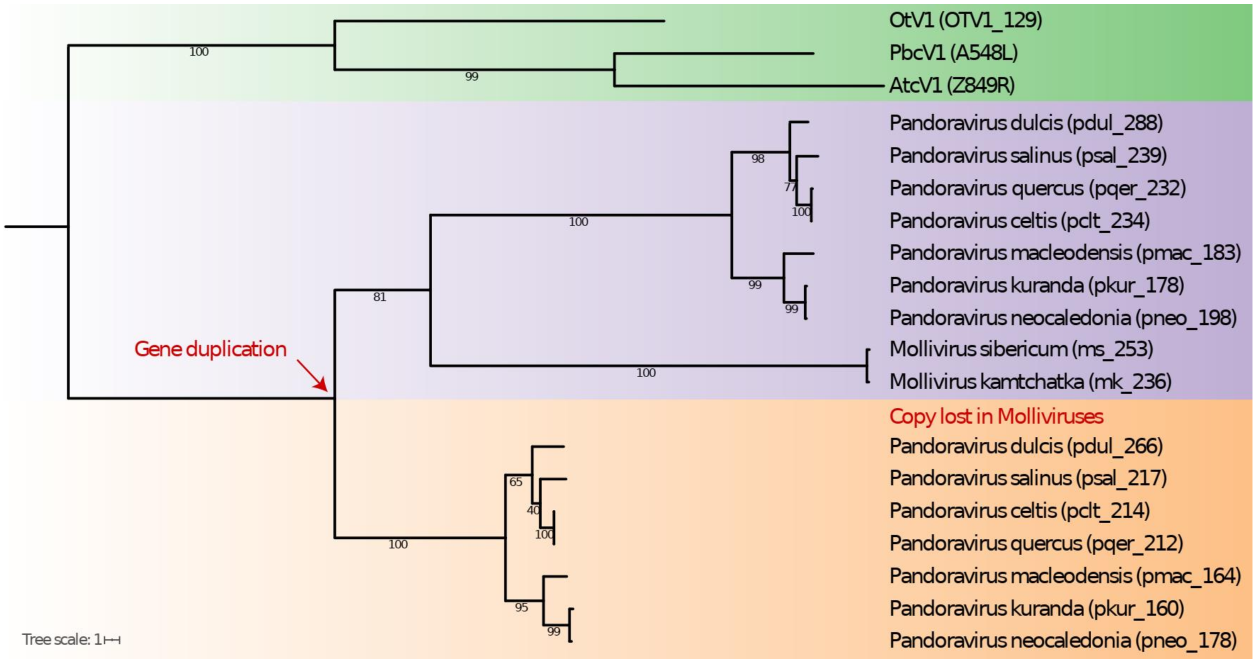

Figure S5. Phylogenetic analysis of the SNF2-like helicases.

(A) Phylogenetic analysis of the SNF2-like helicases in selected members of the *Phycodnaviridae*, mollivirus and pandoravirus. *Emiliania huxleyi* virus 86 (NC_007346.1), Feldmannia species virus (NC_011183.1), *Ectocarpus siliculosus* virus 1 (NC_002687.1), *Ostreococcus tauri* virus 1 (NC_013288.1), *Paramecium bursaria* Chlorella virus 1 (NC_000852.5), *Mollivirus sibericum* (NC_02 7867.1), *Mollivirus kamchatka* (MN812837.1), *Pandoravirus dulcis* (NC_021858.1), *Pandoravirus salinus*

(NC_022098.1), *Pandoravirus quercus* (NC_037667.1), *Pandoravirus macleodensis* (NC_037665.1). and *Pandoravirus kuranda* (ON887157).

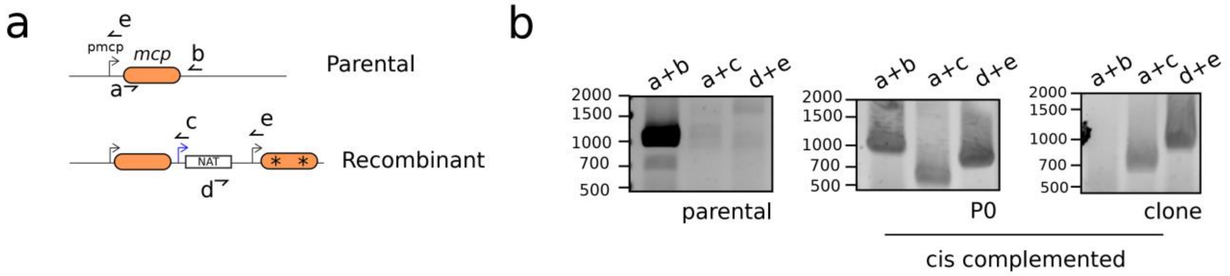

Figure S6. Mollivirus MCP acts as a scaffolding protein for tegument biosynthesis.
(A) Schematic representation of the *mcp* locus and primer annealing sites.
(B) PCR of the genomic locus of the *mcp* demonstrate correct integration and clonality. Integration in the population at the generation 0 (P0, 24hours post transfection) is also shown. Expected size for PCR products: a+b (parental): 1000bp, a+c (recombinant): 650bp, d+e (recombinant): 800bp. PCRs are representative of 2 independent experiments.

Table S1: List of primers used in this study.

ID	Purpose	Sequence
HB17	3'UTR-pU6-(tRNA-guide-scaffold)	CTGGGCGGTGACTAAtctag
HB18	3'UTR-pU6-(tRNA-guide-scaffold)	gttgttccgggtcgactctaG
HB19	convert guide to NotI for clonning	CTGGCCAgcggccgcGttttagagctagaaatagcaag
HB20	convert guide to NotI for clonning	ctaaaacgcggccgcTGGCCAGTATGGGGATC
HB21	guide RBP1 pandoravirus neocal	TCCCCATACTGGCCAgTGACGGCCACCACCGAACCGgttttagagctagaaatagc
HB22	guide RBP1 pandoravirus neocal	ttctagctctaaaacGATTGTGCGCCGCATGTCCGTGGCCAGTATGGGG
HB23	guide RBP1 mollivirus kamch	TCCCCATACTGGCCAAGACGCGCTCGAGATTCTCGgttttagagctagaaatagc
HB24	guide RBP1 mollivirus kamch	ttctagctctaaaacGCTCGAGGTCCACATACCTGTGGCCAGTATGGGG
HB25	guide RBP1 pithovirus siber	TCCCCATACTGGCCAgCGAGGTGACCGGGACATTCGgttttagagctagaaatagc
HB26	guide RBP1 pithovirus siber	ttctagctctaaaacGATGGGTGAGATCATCACCGTGGCCAGTATGGGG
HB27	convert guide to NotI for clonning	gtcctgtcgggtttcgcc

HB28	convert guide to NotI for clonning	cgaaacccgacaggac
HB29	Check genome of RBP1 Queen	GACATTCGGCTATTGTAGATCCT
HB30	Check genome of RBP1 Queen	CAAAAGGTCCAAGACCCTC
HB31	Check genome of RBP1 Queen	GAGAACCATCGCCTTTAGTG
HB32	Check genome of RBP1 Pitho siber	CTTGGAGGTGGTATCCGTC
HB33	Check genome of RBP1 Pitho siber	TTCCTCGTAATCAGCCTCC
HB34	Check genome of RBP1 Pitho siber	CAGATAAGGGCACGAACAAC
HB35	Check genome of RBP1 Pandora neocal	ATGACATCGCCGGTCATCTAC
HB36	Check genome of RBP1 Pandora neocal	GTCCGCTGCCGAGGTG
HB37	Check genome of RBP1 Pandora neocal	TGCCTTGCCGCGCTTG
HB38	Check genome of RBP1 Molli kamtch	GAGGCCTCAACGACCAC
HB39	Check genome of RBP1 Molli kamtch	GATGCCGGACACCACCAC
HB40	Check genome of RBP1 Molli kamtch	CACGGCATGCAGCAGGC
HB49	guide against RFP	TCCCCATACTGGCCAgCTTGGTCACCTTCAGCTTGGgttttagagctagaaatagc
HB50	guide against RFP	ttctagctctaaaacCGATGGTGTAGTCCTCGTTGcTGGCCAGTATGGGG
HB60	Guide NLF3 pando	TCCCCATACTGGCCAg**CCTGGCTCGCTGCACTCTGT**gttttagagctagaaatagc
HB61	Guide NLF3 pando	ttctagctctaaaacCCGAGCGCGTGCGCCAGATCTGGCCAGTATGGGG
HB62	guide MCP molli	TCCCCATACTGGCCAgCAACGACACCGCCGTGAGCCgttttagagctagaaatagc
HB63	guide MCP molli	ttctagctctaaaacTGCGCCGCAGCATCCACATGcTGGCCAGTATGGGG
HB64	Guide Pneo-177	TCCCCATACTGGCCA**GTTGGGCGAGGACATtgacg**gttttagagctagaaatagc
HB65	Guide Pneo-177	ttctagctctaaaacACACCAAGTCCTCGTCGTGCTGGCCAGTATGGGG
HB66	Guide **pneo-585**	TCCCCATACTGGCCAg**CGTTGGCGTTGGTCGTATAG**gttttagagctagaaatagc
HB67	Guide **pneo-585**	ttctagctctaaaacGTGGACGCTCGCCGACCAGTcTGGCCAGTATGGGG
HB68	guide **pneo-972**	TCCCCATACTGGCCAg**CATTGGCCTTGGTCCAGTAG**gttttagagctagaaatagc
HB69	guide **pneo-972**	ttctagctctaaaacCCGACGTGTCGGTGACGTTGcTGGCCAGTATGGGG
HB76	To generate Cas9 to integrate in RFP locus	AGAATTCACGCGTCCGGAGGCATCGTGGAACAGTACGAGCG
HB77	To generate Cas9 to integrate in RFP locus	ATATAGATCTaggcctGCTGACTAATTGAGATGCATGC

HB78	To generate Cas9 to integrate in RFP locus	ATTAGTCAGCaggcctAGATCTATATGCAACCCTGTGACG
HB79	To generate Cas9 to integrate in RFP locus	CGCTGCACCTAGGGGAGAGGCTTGGCGGTCTGGGTGCC
HB84	MCP1-RFP	TCTCACTAAGACATAATGACGGACCACCTTCTGGC
HB85	MCP1-RFP	ccTCGGAGGAGGCCATCGCTCGGCACACGTGGTTG
HB86	RFP-MCP1	CACTCCACCGGCGCCATGACGGACCACCTTCTGGC
HB87	RFP-MCP1	CCGGGTCGACTCTAGTTACGCTCGGCACACGTG
HB88	Check genome of MCP Molli	cggctttcgtttcgtccatcgtgc
HB89	Check genome of MCP Molli	CGACGGCCGTCGCGTCG
HB90	Check genome of NLF3 Pandora neocal	gtctgcccttgtcctctgttgtc
HB91	Check genome of NLF3 Pandora neocal	GGTCCCGACAGGCCGGTG
HB92	Check genome of Pneo-177 Pandora neocal	ctcccagatgcgtcgtgctcc
HB93	Check genome of Pneo-177 Pandora neocal	ccggcgaggcagcgacc
HB94	Check genome of pneo-585 Pandora neocal	ATGCAACGACCTGTAGCGACCG
HB95	Check genome of pneo-585 Pandora neocal	ggcgcccgccatatggac
HB96	Check genome of pneo-972 Pandora neocal	gtccagATGGGTATGCGTGCTCG
HB97	Check genome of pneo-972 Pandora neocal	gtccggtgtcagcacttaccaagc
HB98	Mutations MCP molli for complementation	tGGgGCtAGaCCAGCGTCGACCAGAAC
HB99	Mutations MCP molli for complementation	gAGaCTgACcGCGGTGTCGTTGATGGC
HB100	Mutations MCP molli for complementation	ACCGCgGTcAGtCTcGCCGACATCTCGCCGGCG
HB101	Mutations MCP molli for complementation	GCTGGTCTAGCCCCACAGCATCCACATGTAGAAGATGATG
HB102	guide CS1-3 A cast	TCCCCATACTGGCCAGAGAACGCGTGCGCCTTCGCgttttagagctagaaatagc
HB103	guide CS1-3 A cast	ttctagctctaaaacgccgtcaagatcctggccacTGGCCAGTATGGGG
HB104	guide pneo182 pandora necoal	TCCCCATACTGGCCAgACTCGCCGGGCACGTGATAGgttttagagctagaaatagc
HB105	guide pneo182 pandora necoal	ttctagctctaaaacCTAACGGCGCCGCAGCCAGCTGGCCAGTATGGGG
HB126	HR MCP molli	TCTCACTAAGACATAtgCGAAGCCATGCCAGGACAG
HB127	HR MCP molli	CCCTTGCTCACCATAATGGCATTGTTGAAGCGGTGGg
HB128	HR MCP molli	GCGTGGTACCTCTAGCCAGCGTCGACCAGAACTC

HB129	HR MCP molli	CCGGGTCGACTCTAGaGGACACGGCAAGGCAAAAC
HB134	reverse primer in RFP (check clonality)	CCAGCCCATGGTCTTCTTCTGC
HB135	CS1-genotyping	GTTCGTGCAGACGCCGCAG
HB136	CS1-genotyping	gcTCGGCGAAGAAGACGAAGAC
HB137	CS1-genotyping	GCGAGGAGGACCGCATCGAG
HB138	CS1-genotyping	GCAGATGCGGTGGGTGAGG
HB139	CS2-genotyping	Agcagcgcgacaagatgctc
HB140	CS2-genotyping	Agatgcggtggcacaggtgg
HB141	CS2-genotyping	gtgctgcgcaagggccag
HB142	CS2-genotyping	tgtagaactgggcccaccag
HB143	CS3-genotyping	TGCCCTGGTTCTACAACGAG
HB144	CS3-genotyping	TGAAATTGGACGGGCGCTGG
HB173	qPCR 5 end pneo genome	TCCTTTTTCCCACCATCCC
HB174	qPCR 3 end pneo genome	TTCTCCCATTCATTCAAACCC
HB175	qPCR 3 end pneo genome	TAGCATAGCATCACCGTCC
HB182	genotyping MCP KO	cgacccgtggctctgtcaac
HB183	genotyping MCP KO	caccaccaacatcatcactatc
HB184	genotyping pneo182 KO	gtctcctccacaccatattg
HB185	genotyping pneo182 KO	cagaagagacaccaccattg
HB199	Promoter pneo650 in for NAT	CTTTTGCAAAAAGCTtCGGCTCTGCAGTTGATCTC
HB200	Promoter pneo650 in for NAT	CAGAAGAATCAAGCTGATGATGGTTGCGGTTGC
HB201	3-UTRP in NAT pneo640	gcggccgcGATATCGCGGGACTCTGGGG
HB202	3-UTRP in NAT pneo640	TTAGGGGCAGGGCATGCTC
HB203	3-UTRP in NAT pneo640	ATGCCCTGCCCCTAATAAGCGGCGCCGTGCCGC
HB204	3-UTRP in NAT pneo640	CGATATCgcggccgcTGTGCCTTAGAGACATTTTGTG
HB213	guide pneo62	TCCCCATACTGGCCAGGACCGCAGCGGGTCCATGGgttttagagctagaaatagc
HB214	guide pneo62	ttctagctctaaaacCGACACTGCAACGCTCGTTTcTGGCCAGTATGGGG
HB215	guide pneo350	TCCCCATACTGGCCAgTGACGTCGTGCGCCCCTCCGgttttagagctagaaatagc
HB216	guide pneo350	ttctagctctaaaacAGGCCGACCAGTACCCGATCTGGCCAGTATGGGG
HB217	guide pneo450	TCCCCATACTGGCCAgTGTCTACGCCCATCTGGACGgttttagagctagaaatagc
HB218	guide pneo450	ttctagctctaaaacTCGCAAATGGCTGCGACATGcTGGCCAGTATGGGG
HB219	guide pneo518	TCCCCATACTGGCCAgCCCGTGCAGTGCTTCACGTGgttttagagctagaaatagc
HB220	guide pneo518	ttctagctctaaaacCCAGCGAGCAGCGCAAGCGCTGGCCAGTATGGGG

HB221	guide pneo959	TCCCCATACTGGCCAgAATGTCGGGCCTGAGACTCGgttttagagctagaaatagc
HB222	guide pneo959	ttctagctctaaaacCAGCGATTTTGCCGGCCTCGcTGGCCAGTATGGGG
HB243	guide pneo178	TCCCCATACTGGCCAgCGTCGGCGAGTACACGATGGgttttagagctagaaatagc
HB244	guide pneo178	ttctagctctaaaacCACGGCGATAGACGCAGAGAcTGGCCAGTATGGGG
HB245	guide pneo179	TCCCCATACTGGCCAgCTCGTGGCCAAGTGGCTCAGgttttagagctagaaatagc
HB246	guide pneo179	ttctagctctaaaacAGGCGCATCGCCTATCGATTcTGGCCAGTATGGGG
HB247	guide pneo181	TCCCCATACTGGCCAgTTTGAGCGCGCCTTTGGCATgttttagagctagaaatagc
HB248	guide pneo181	ttctagctctaaaacTCTGCAGCGAGCGACACAGCcTGGCCAGTATGGGG
HB249	guide pneo537	TCCCCATACTGGCCAgTGACGTGTCCAATCTCATAGgttttagagctagaaatagc
HB250	guide pneo537	ttctagctctaaaacCCGCCGTGCGCAAGGCCATCTGGCCAGTATGGGG
HB251	guide pneo557	TCCCCATACTGGCCAgAAAGGCGTCAAACTCTGGGGgttttagagctagaaatagc
HB252	guide pneo557	ttctagctctaaaacCGCAGCAGGAACGCCAGAGAcTGGCCAGTATGGGG
HB253	guide pneo575	TCCCCATACTGGCCAgACAGTTTGCCGATCCGGCGGgttttagagctagaaatagc
HB254	guide pneo575	ttctagctctaaaacCAGACAGAGACGACACCAACTGGCCAGTATGGGG
HB265	3 UTR MCP cis-complementation	TAAGGCACAGCGGCCACTCGTCCTCCCGTTTCCC
HB266	3 UTR MCP cis-complementation	CGCGATATCGCGGCCgcCGGCCACATCGTGCAGCAG
HB267	5 UTR MCP cis-complementation	TTTGTTGCGCGAATTcCAGGTGCAGCACCACTCC
HB268	5 UTR MCP cis-complementation	aaaatAAGAACAAGAgggcccTTACGCTCGGCACACGTG
HB295	Check integration MCP 3' end	CGGCAACCAGATCATGGTCATC
HB296	Check integration MCP 3' end	gtcACTTCCTGCATACTTGTGG
HB312	guide pneo 100	TCCCCATACTGGCCAACAGGCCATCGGCATCTGTTgttttagagctagaaatagc
HB313	guide pneo 100	ttctagctctaaaacCAAGCGGACATATCGCCCTTTGGCCAGTATGGGG
HB314	guide pneo 150	TCCCCATACTGGCCAAGACGCGGAAAGGTCTTCTGgttttagagctagaaatagc
HB315	guide pneo 150	ttctagctctaaaacCGACGCAACACAGACCGTGAcTGGCCAGTATGGGG
HB316	guide pneo 243	TCCCCATACTGGCCAGGTCGCCACGGGTCCGGTTGgttttagagctagaaatagc
HB317	guide pneo 243	ttctagctctaaaacCGCAAGGTCCGTCCGAGACCTGGCCAGTATGGGG
HB318	guide pneo 289	TCCCCATACTGGCCAATAGTAGCGCGAGATGGCGTgttttagagctagaaatagc
HB319	guide pneo 289	ttctagctctaaaacAAGGTCAACGTGGGCACCATTGGCCAGTATGGGG
HB320	guide pneo 523	TCCCCATACTGGCCAgCCATGGTGGCCTATGTGTGGgttttagagctagaaatagc
HB321	guide pneo 523	ttctagctctaaaacCGACCCGGCAGCATGCGGTCTGGCCAGTATGGGG
HB322	guide pneo 528	TCCCCATACTGGCCAgCAAGTTGTCGTATCGCCGGGgttttagagctagaaatagc

HB323	guide pneo 528	ttctagctctaaaacCATTCGCCCCGGCACAGTGCTGGCCAGTATGGGG
HB324	guide pneo 533	TCCCCATACTGGCCAgTCTTTGTCGACAATCGACCGgttttagagctagaaatagc
HB325	guide pneo 533	ttctagctctaaaacCCACCATGTCGCCCCACCCGcTGGCCAGTATGGGG
HB326	guide pneo 600	TCCCCATACTGGCCAgCAAATGAAGGCCCACAATGGgttttagagctagaaatagc
HB327	guide pneo 600	ttctagctctaaaacCTATAGGTCGCCACAATATCTGGCCAGTATGGGG
HB328	guide pneo 177	TCCCCATACTGGCCAgCGACGACGGAGCCACGTAAGgttttagagctagaaatagc
HB329	guide pneo 177	ttctagctctaaaacTTGGTGTTTGCCCCGCCGCAcTGGCCAGTATGGGG
HB330	guide pneo 801	TCCCCATACTGGCCAaATGGGGCGAAATCGACCCGgttttagagctagaaatagc
HB331	guide pneo 801	ttctagctctaaaacCGGCACGCGGTGGTCATATGcTGGCCAGTATGGGG
HB332	guide pneo 650	TCCCCATACTGGCCA**GTCTGCCTTCCAGAACAACCT**gttttagagctagaaatagc
HB333	guide pneo 650	ttctagctctaaaac**CCGGCGGAAACTACGCGGTGC**TGGCCAGTATGGGG
HB334	guide pneo 700	TCCCCATACTGGCCA**GCGAGGGCCATCATCGCGTCG**gttttagagctagaaatagc
HB335	guide pneo 700	ttctagctctaaaacCGGCAATCACACGAGCCAACTGGCCAGTATGGGG
HB336	guide pneo 750	TCCCCATACTGGCCA**GAGAGCCATCGCAATGTGTCG**gttttagagctagaaatagc
HB337	guide pneo 750	ttctagctctaaaac**CTGGCAATGAGGTGCCCGATC**TGGCCAGTATGGGG
HB338	guide pneo 850	TCCCCATACTGGCCAG**GACGCTAGTACCGATCCATGG**gttttagagctagaaatagc
HB339	guide pneo 850	ttctagctctaaaac**CGTGGCATCACAGACAGTCC**TGGCCAGTATGGGG
HB340	guide pneo 900	TCCCCATACTGGCCAG**GTCTCGCGGCGCGCAGATTGT**gttttagagctagaaatagc
HB341	guide pneo 900	ttctagctctaaaacCGAGTCTCGGCAGCGCCATTCTGGCCAGTATGGGG
HB342	guide pneo 950	TCCCCATACTGGCCAGCTTTGTCGCGAGACTCTTGGgttttagagctagaaatagc
HB343	guide pneo 950	ttctagctctaaaac**CGTAGATCGTGGTCCCGTCGC**TGGCCAGTATGGGG
HB390	genotype pneo100	GGCGCTATCGGCCAC
HB391	genotype pneo100	Aaagcgatctgcttactcc
HB392	genotype pneo150	TGACGAGTGCCTGGCAGC
HB393	genotype pneo150	CCTTGGCCCACGAGGCC
HB394	genotype pneo575	gctagagaggcacaccggg
HB395	genotype pneo575	GCCGTTCTTTGACCACAAGGG
HB414	genotyping pneo243	GCGCCTGCGATCTGACGACG
HB415	genotyping pneo243	GGAAGGGAGCGCACGCGAC
HB416	genotyping pneo289	GCCGACGGTCAGCGAG
HB417	genotyping pneo289	TAGGTGGCAGGCGCGTC

HB418	genotyping pneo523	TGCCGCGGCGTGAGCGTG
HB419	genotyping pneo523	CCACACGCTGGCCAGGTTGG
HB420	genotyping pneo700	GACAACAACGACGCCAAAG
HB421	genotyping pneo700	TCGACATCCTCAATGTCGGC
HB422	genotyping pneo801	Ctctccgtgtgcgcctacc
HB423	genotyping pneo801	GGCCATGGATGTGCAACGCG
HB424	genotyping pneo600	CCGCTGCTCGATCGGGTCG
HB425	genotyping pneo600	GCCATGTGCCCGAGATGCGG
HB426	genotyping pneo750	GCGGCCACTGGTCGAC
HB427	genotyping pneo750	CACGGACATCAGCCATCG
HB428	genotyping pneo850	CCCAAGTAGCGTCAAAGGG
HB429	genotyping pneo850	TCGTCGTCATCATCAACGTC
HB430	genotyping pneo900	GGCGCGCTGGTCTCTGAG
HB431	genotyping pneo900	GTACAGGCAGTCAAGACTGC
HB432	genotyping pneo950	GTGTGAGGAGAGATGCGGTC
HB433	genotyping pneo950	CTCGGCGTTGCGCAGG
HB434	genotyping pneo533	TTTGCCACGGCGTGGGAG
HB435	genotyping pneo533	AGCGTGTGTCGCGAAGATAG
HB436	genotyping pneo518	Tctcgcaggctctgcccgac
HB437	genotyping pneo518	cggcgtgctgcgcgcatc
HB438	genotyping pneo450	aatccacattgccgcggag
HB439	genotyping pneo450	AGACGACAAAGTCCCACATG
HB440	genotyping pneo557	GCTCACCGCCGAGGCCTACC
HB441	genotyping pneo557	GCCTCGGCCAACTCGGCGC
HB448	guide pneo 77	TCCCCATACTGGCCAGCCCGCCTATTAGACGATCGgttttagagctagaaatagc
HB449	guide pneo 77	ttctagctctaaaacCGGCGGTTGGAGGCTCCATGcTGGCCAGTATGGGG
HB450	guide pneo 153	TCCCCATACTGGCCAgCGTGGCCAGCGGCACCAACGgttttagagctagaaatagc
HB451	guide pneo 153	ttctagctctaaaacCCAAACTCGTGCCACCGTCGcTGGCCAGTATGGGG
HB452	guide pneo 228	TCCCCATACTGGCCAgTTTCGCCTCTGGGATCGATGgttttagagctagaaatagc
HB453	guide pneo 228	ttctagctctaaaacCCAAGCATCGCGCTGCCAAAcTGGCCAGTATGGGG
HB454	guide pneo 238	TCCCCATACTGGCCAgCGACGGGACGCTCATCATCGgttttagagctagaaatagc
HB455	guide pneo 238	ttctagctctaaaacCAGTTGGACCTGCGCAAGTTTGGCCAGTATGGGG

HB456	guide pneo 248	TCCCCATACTGGCCAGGTGGTGCTGTACACAACAGgttttagagctagaaatagc
HB457	guide pneo 248	ttctagctctaaaacCAACAAGCACGAGACCGAGGcTGGCCAGTATGGGG
HB458	guide pneo 251	TCCCCATACTGGCCAgCGTGTGGTAATACCGCCCGTgttttagagctagaaatagc
HB459	guide pneo 251	ttctagctctaaaacCGAACCGACGGTGCGCGCCAcTGGCCAGTATGGGG
HB460	guide pneo 254	TCCCCATACTGGCCAGGACTATACGGTCTCGACGGgttttagagctagaaatagc
HB461	guide pneo 254	ttctagctctaaaacCTCATGGGCGCGCTGCCTTGcTGGCCAGTATGGGG
HB462	guide pneo 264	TCCCCATACTGGCCAgTGACGCGGCGCTTTGTCATGgttttagagctagaaatagc
HB463	guide pneo 264	ttctagctctaaaacCGACGATGGCCTCGCCGTGGcTGGCCAGTATGGGG
HB464	guide pneo 301	TCCCCATACTGGCCAGTTGGAGAATGGGATGATGGgttttagagctagaaatagc
HB465	guide pneo 301	ttctagctctaaaacCTGTGATGCCCCTTACGACGcTGGCCAGTATGGGG
HB466	guide pneo 315	TCCCCATACTGGCCAAAAGGACGTCGCCTGGCCGGgttttagagctagaaatagc
HB467	guide pneo 315	ttctagctctaaaacCTGCCCGAGAGAAACACATTTGGCCAGTATGGGG
HB468	guide pneo 321	TCCCCATACTGGCCAgTATCGGCAGATCATCATCGGgttttagagctagaaatagc
HB469	guide pneo 321	ttctagctctaaaacCCAGTCACGGGCGCGCCTTAcTGGCCAGTATGGGG
HB470	guide pneo 325	TCCCCATACTGGCCAgTGACGTGGGCCTCCCACTTGgttttagagctagaaatagc
HB471	guide pneo 325	ttctagctctaaaacCATGGGACCGCGGGGCGTCTTGGCCAGTATGGGG
HB472	guide pneo 365	TCCCCATACTGGCCAGAATGGGCCGGACATTGCCGgttttagagctagaaatagc
HB473	guide pneo 365	ttctagctctaaaacCGATGAACCAGCGGTAGTTGcTGGCCAGTATGGGG
HB474	guide pneo 383	TCCCCATACTGGCCAgTGATTGCGAGGGCACGTCGGgttttagagctagaaatagc
HB475	guide pneo 383	ttctagctctaaaacCGAATGTGCGACGAGCAACAcTGGCCAGTATGGGG
HB476	guide pneo 385	TCCCCATACTGGCCAGTGCGCCAGCGAGCGCATAGgttttagagctagaaatagc
HB477	guide pneo 385	ttctagctctaaaacCCTGGCGCTCTCTGGCCAACTGGCCAGTATGGGG
HB478	guide pneo 417	TCCCCATACTGGCCAGATGGCCAACGCGCTCGTTGgttttagagctagaaatagc
HB479	guide pneo 417	ttctagctctaaaacCCAAAGGACCGACGCCAAAGcTGGCCAGTATGGGG
HB480	guide pneo 459	TCCCCATACTGGCCAgCTGCTCCAAGGCTATCCTGGgttttagagctagaaatagc
HB481	guide pneo 459	ttctagctctaaaacCCAACTTCCTCGCAGACACTTGGCCAGTATGGGG
HB482	genotype 178	GCACTGTGCAGACGCGCGTC
HB483	genotype 178	CAACACGACTCGTGTCGTGC
HB484	genotype 179	ATGCAGTGCCGCAAGAACCC
HB485	genotype 179	ATTGGCGCGTAACCTGCGG
HB486	genotype 181	CCATGATCTTCCTAGACGCG
HB487	genotype 181	gcaaCTAGAGGGGCGAGTC
HB488	genotype 350	GGCGCCTACCCCATACCGTC

HB489	genotype 350	GTGCGGACGGTCGGCGTC
HB490	cis complementation MCP	ACTCGTCCTCCCGTTTCC
HB491	cis complementation MCP	TGTGCCTTAGAGACATTTTGTG
HB492	cis complementation MCP	CAAAATGTCTCTAAGGCACATTTGTCCGTCTGTCCACTTTGG
HB493	cis complementation MCP	GGTCCGTCATCGTTTCTTTGCTCGCTGGGG
HB494	cis complementation MCP	CAAAGAAACGATGACGGACCACCTTCTGGC
HB495	cis complementation MCP	GGGGAAACGGGAGGACGAGTTTACGCTCGGCACACGTG
HB502	genotype pneo153	GCTGCGCACACGCATCGATC
HB503	genotype pneo153	GTCGCAGCGCGCCTTGC
HB504	genotype pneo228	TTCGGCCTACCTGGCCG
HB505	genotype pneo228	GCCGGCATGCAATGCCAC
HB506	genotype pneo238	CCTGCACCAGGGCACCTC
HB507	genotype pneo238	CAGTGGACGATGCCCCACG
HB508	genotype pneo248	CTCGTGGCACACACCGCC
HB509	genotype pneo248	CAGCGCCTTCGAGCGCC
HB510	genotype pneo251	GAGCAGCGGAGCGATGCC
HB511	genotype pneo251	CGTCCCTTGTGCGTCGGAC
HB512	genotype pneo254	GCGTGCGCCTGCGTCATTTC
HB513	genotype pneo254	GTGCGATAGTCGGGCGCAC
HB514	genotype pneo264	GCCTACTATGGCGTCGAGAG
HB515	genotype pneo264	ACTGCTCGGGAAAGTCCATG
HB516	genotype pneo301	GATGGACGAGGACGACGATG
HB517	genotype pneo301	CGAACTCGAGCACGAACC
HB518	genotype pneo315	GAGTTTGCCAGCGTCCGG
HB519	genotype pneo315	GCGCATTCGATTGAGCACGG
HB520	genotype pneo321	CCCAACGACACGCGGCTC
HB521	genotype pneo321	CGAGCCGCAAGGCGTTG
HB522	genotype pneo325	CGACCGATGTCAACCGCG
HB523	genotype pneo325	CATGTTGAGGCACGCGCAC

HB524	genotype pneo365	CCCGCATCTCAACACGCAG
HB525	genotype pneo365	TGGCGTGGTGCATGACGATG
HB526	genotype pneo383	CTGCAGACTCTGCGTTGGCG
HB527	genotype pneo383	CGTCGTGCACCACCTTGGTC
HB528	genotype pneo385	GGAGACCACCTAATGGCCG
HB529	genotype pneo385	ACGTCACATGGCATCGATCC
HB530	genotype pneo417	GGAACCGCGATAGCACCACG
HB531	genotype pneo417	GGTCGCGCTCAGAGAGTAC
HB532	genotype pneo459	CATCGTCGAGCGCCTCAC
HB533	genotype pneo459	CGACAGGTCGGATCCGAC
HB534	guide pneo 187	TCCCCATACTGGCCAgCACGTGCCTCGGCATTTGGGgttttagagctagaaatagc
HB535	guide pneo 187	ttctagctctaaaacCGATAGTAGTGCCGACGAAAcTGGCCAGTATGGGG
HB536	guide pneo 198	TCCCCATACTGGCCAgCAAGGTACTGCGCCTCTTGGgttttagagctagaaatagc
HB537	guide pneo 198	ttctagctctaaaacCTGCTATCGCCATCCCCAGCTGGCCAGTATGGGG
HB538	guide pneo 221	TCCCCATACTGGCCAGTAGGGAAAGCTGCTCACGGgttttagagctagaaatagc
HB539	guide pneo 221	ttctagctctaaaacCCGCCCAACCAGTACCGAGCTGGCCAGTATGGGG
HB540	guide pneo 231	TCCCCATACTGGCCAgCGAGCGCATGGGGAGCGTTGgttttagagctagaaatagc
HB541	guide pneo 231	ttctagctctaaaacCAAGAATGCGTCGGTACGTGcTGGCCAGTATGGGG
HB542	guide pneo 250	TCCCCATACTGGCCAgTGGCGACGGCGGTCGCGCTGgttttagagctagaaatagc
HB543	guide pneo 250	ttctagctctaaaacCCATCGATCCCACCAACATCTGGCCAGTATGGGG
HB544	guide pneo 261	TCCCCATACTGGCCAgAATCTCATTGCGACCCATGGgttttagagctagaaatagc
HB545	guide pneo 261	ttctagctctaaaacCACCGCGCCCGACCGCGTATcTGGCCAGTATGGGG
HB546	guide pneo 276	TCCCCATACTGGCCAgCAAGGGCGTGGCGATCGTCGgttttagagctagaaatagc
HB547	guide pneo 276	ttctagctctaaaacCCAGCGAGCGGCCGCACCATcTGGCCAGTATGGGG
HB548	guide pneo 304	TCCCCATACTGGCCAgCATGTGACCCACGCCGTCGGgttttagagctagaaatagc
HB549	guide pneo 304	ttctagctctaaaacAGATGATGTGCCTGGCCACGcTGGCCAGTATGGGG
HB550	guide pneo 313	TCCCCATACTGGCCAgTGAGGCCGACTGGAGGTCATgttttagagctagaaatagc
HB551	guide pneo 313	ttctagctctaaaacCAAAGCAGCACGCACGCTCCTGGCCAGTATGGGG
HB552	guide pneo 322	TCCCCATACTGGCCAgCCACTTTGCGGCCCTGTTAGgttttagagctagaaatagc
HB553	guide pneo 322	ttctagctctaaaacCTGCCGATCGGCCATCGTCCTGGCCAGTATGGGG
HB554	guide pneo 342	TCCCCATACTGGCCAGGTCGCTCACGCGCTCGTCGgttttagagctagaaatagc

HB555	guide pneo 342	ttctagctctaaaacCCATGCACACCGACGAGATCTGGCCAGTATGGGG
HB556	guide pneo 344	TCCCCATACTGGCCAgCGCCGGGCGCAAGTTTGAAGgttttagagctagaaatagc
HB557	guide pneo 344	ttctagctctaaaacCGATGAGATCGTCGGCCTCGcTGGCCAGTATGGGG
HB558	guide pneo 407	TCCCCATACTGGCCAgTTTCGATCCAGCGTTCGGGGgttttagagctagaaatagc
HB559	guide pneo 407	ttctagctctaaaacCCGACCTGCTCGACAAGATAcTGGCCAGTATGGGG
HB560	guide pneo 414	TCCCCATACTGGCCAgAGAGTGCGTGGCCTTTATGGgttttagagctagaaatagc
HB561	guide pneo 414	ttctagctctaaaacCGAACAGCTGCACGCCGAGCTGGCCAGTATGGGG
HB562	guide pneo 425	TCCCCATACTGGCCAgATACTCTTGGCGGATGCCGGgttttagagctagaaatagc
HB563	guide pneo 425	ttctagctctaaaacCAGACGATCAGAGCCAGACAcTGGCCAGTATGGGG
HB564	guide pneo 429	TCCCCATACTGGCCAgCGAATGGACGGCCACCCTCGgttttagagctagaaatagc
HB565	guide pneo 429	ttctagctctaaaacCCAATATGGCCGAGGCCATCTGGCCAGTATGGGG
HB566	guide pneo 443	TCCCCATACTGGCCAgCGTGCGCGGTTCTACGCTTGgttttagagctagaaatagc
HB567	guide pneo 443	ttctagctctaaaacCAAGTATGCAGCGGCACGCGcTGGCCAGTATGGGG
HB568	guide pneo 544	TCCCCATACTGGCCAgAAAAGAATCGCGTCTGTTGGgttttagagctagaaatagc
HB569	guide pneo 544	ttctagctctaaaacCCATTGATCGCGTGGCGTCGcTGGCCAGTATGGGG
HB572	qPCR molli	TGATGGATGGATACAGCGAG
HB573	qPCR molli	GAGAAGGAGGAGAAGGAAGAG
HB576	genotype 187	GCCAACACTCGACGCGAC
HB577	genotype 187	GAGGTCAAGCGCATTTGTG
HB578	genotype 198	GACGCAACGGACGAGCAG
HB579	genotype 198	CACAAACTGGCGTGTCCATC
HB580	genotype 221	CGCGCACTGCCATACTCG
HB581	genotype 221	GCCTGCGCCATCATTTGC
HB582	genotype 231	GCCCAGTCAACACACACG
HB583	genotype 231	ACAGTTCTGCGACCATGTG
HB584	genotype 250	TCAGGCCTCACGCGAGC
HB585	genotype 250	TTGCCGCCTGATCGGATG
HB586	genotype 261	GGCAACAATGACAATGAGCATG
HB587	genotype 261	CATGCTGCTTATGCGGTCG
HB588	genotype 276	CAGTGCAACGCGTCGAC
HB589	genotype 276	TATGTAGTCGGCCACGAG
HB590	genotype 304	CATGCACTTTGACATGGGC
HB591	genotype 304	ACACAGCTGGTAGGTGAC
HB592	genotype 313	TGGCGGTGGCGATGATG
HB593	genotype 313	CAAACATCGCACGACGATCG
HB594	genotype 322	CTTTCAGTGCCCGGAGC
HB595	genotype 322	CGCGTATAATAGAGCCAGGC

HB596	genotype 342	TACGCGCTCATGGAGACC
HB597	genotype 342	GAACCACAAGAGCAGGTAGG
HB598	genotype 344	CAACCCGGTGAGCGTGAG
HB599	genotype 344	CGCAGCGGCGTGAGAATG
HB600	genotype 407	CGTCGACGACATTGCGCG
HB601	genotype 407	CTGCCGTAGCCGCAGTG
HB602	genotype 414	CAAGGAACGCCAACAGACC
HB603	genotype 414	GGTGGGGTCGACCGAAGC
HB604	genotype 425	ATGACCGAGACGCATCGC
HB605	genotype 425	TGCGGCGAGTTGAGACCG
HB606	genotype 429	CTCGACAATGTGCACCACG
HB607	genotype 429	GGCTTGCACGCCAGGTTC
HB608	genotype 443	ACGCCGAGCGCATCGTG
HB609	genotype 443	CGCCATTCTGCCAGCGTC
HB610	genotype 544	CGTCGACGCGCCAGACAG
HB611	genotype 544	GTAGGCGGCCTCTTCAACG
HB612	genotype mk236	GAGCCCCAAGGTCAGTCC
HB613	genotype mk236	CCAGTCGTTGCATGCCTG
HB614	genotype 875	ACGCACCTCCACTATGAGG
HB615	genotype 875	GAGGATGCCGATCTGGACG
HB616	genotype 925	CGTGCCTTTCCAAGTGCG
HB617	genotype 925	CGTTGCTGGTCGGTTCAG
HB618	genotype 1064	AGCCTCTGCGTGGTCGAC
HB619	genotype 1064	CAGTGCGTGCCGGTAGTG
HB740	Endogenous taging Rbp2 pneo add HA	TTTGTTGCGCGAATTcTACCCATACGATGTTCCTGAC
HB741	Endogenous taging Rbp2 pneo add HA	aaaatAAGAACAAGAGTTAAGCGTAATCTGGAACGTC
HB742	Endogenous taging Rbp2 pneo add FLAG	TTTGTTGCGCGAATTCCATATGTCAGACTACAAAGACCATG
HB743	Endogenous taging Rbp2 pneo add FLAG	aaaatAAGAACAAGATTACTTGTCATCGTCATCCTTG
HB744	5HR FLAG+HA	TTTGTTGCGCGAATTCAAGAACGCGCCCGTCGTCG
HB745	5HR FLAG	GTAGTCTGACATATGATTGTCGGCATTGTCGTGATCG
HB746	5HR HA	AACATCGTATGGGTAATTGTCGGCATTGTCGTGATCG
HB747	3HR	tgtctcTAAGGCACACCGCCGGTGCTCTCCGAATG
HB748	3HR	CGCGATATCGCGGCCgcCTCTGGCCGCCCCACGCC
HB749	gRNA pneoRbp1	TCCCCATACTGGCCAGGTGGTGGCGATGATTACGGgttttagagctagaaatagc
HB750	gRNA pneoRbp1	ttctagctctaaaacCCACGCGGCCCGACGCCATCTGGCCAGTATGGGG
HB751	gRNA pneoRbp1	TCCCCATACTGGCCAGGTCGGGTGCGGCTTCCTCAgttttagagctagaaatagc
HB752	gRNA pneoRbp1	ttctagctctaaaacATGTCAACGACCGCCACTTTcTGGCCAGTATGGGG

Table S2: List of constructs generated in this study.

ID	Purpose	Addgene ID
vHB4	GFP-Cas9/guidesRBP1 queen	
vHB5	GFP-Cas9-NLS/guidesRBP1 queen	
vHB6	lowGFP-Cas9/guidesRBP1 queen	
vHB7	GFP-Cas9/NotI site	
vHB8	GFP-Cas9-NLS/NotI site	193454
vHB9	lowGFP-Cas9/NotI site	
vHB11	GFP-Cas9-NLS/guidesRBP1 Molli kamtch	
vHB12	GFP-Cas9/guidesRBP1 Pitho siber	
vHB13	lowGFP-Cas9/guidesRBP1 Pitho siber	
vHB16	GFP-Cas9-NLS/guidesRBP1 Pandora neocal	193455
vHB17	GFP-Cas9-NLS/guides Red Fluor Prot	
vHB18	GFP-Cas9-NLS/guides NLF3 Pandora neocal	
vHB19	GFP-Cas9-NLS/guides MCP Molli kamtch	
vHB20	GFP-Cas9-NLS/guides Pneo-177 Pandora neocal	
vHB21	GFP-Cas9-NLS/guides pneo-585 Pandora neocal	
vHB22	GFP-Cas9-NLS/guides pneo-972 Pandora neocal	
vHB27	GFP-Cas9-NLS/guidesRFP for homolgous recombination in RFP	
vHB29	MCP–RFP nouseo selection (molli)	
vHB30	RFP-MCP nouseo selection (molli)	
vHB32	GFP-Cas9-NLS/guides pneo-182 Pandora neocal	
vHB55	GFP-Cas9-NLS/guides pneo-62 Pandora neocal	
vHB56	GFP-Cas9-NLS/guides pneo-350 Pandora neocal	
vHB57	GFP-Cas9-NLS/guides pneo-450 Pandora neocal	
vHB58	GFP-Cas9-NLS/guides pneo-518 Pandora neocal	
vHB59	GFP-Cas9-NLS/guides pneo-959 Pandora neocal	
vHB66	3'UTRpneo480-pneo650-vc241 KO	193456
vHB72	GFP-Cas9-NLS/guides pneo-178 Pandora neocal	
vHB73	GFP-Cas9-NLS/guides pneo-179 Pandora neocal	
vHB74	GFP-Cas9-NLS/guides pneo-181 Pandora neocal	
vHB75	GFP-Cas9-NLS/guides pneo-537 Pandora neocal	
vHB76	GFP-Cas9-NLS/guides pneo-557 Pandora neocal	
vHB77	GFP-Cas9-NLS/guides pneo-575 Pandora neocal	
vHB93	GFP-Cas9-NLS/guides pneo-523 Pandora neocal	
vHB94	GFP-Cas9-NLS/guides pneo-528 Pandora neocal	
vHB95	GFP-Cas9-NLS/guides pneo-600 Pandora neocal	
vHB96	GFP-Cas9-NLS/guides pneo-700 Pandora neocal	
vHB97	GFP-Cas9-NLS/guides pneo-750 Pandora neocal	
vHB98	GFP-Cas9-NLS/guides pneo-850 Pandora neocal	

vHB99	GFP-Cas9-NLS/guides pneo-900 Pandora neocal
vHB100	GFP-Cas9-NLS/guides pneo-243 Pandora neocal
vHB101	GFP-Cas9-NLS/guides pneo-950 Pandora neocal
vHB102	GFP-Cas9-NLS/guides pneo-533 Pandora neocal
vHB103	GFP-Cas9-NLS/guides pneo-100 Pandora neocal
vHB104	GFP-Cas9-NLS/guides pneo-650 Pandora neocal
vHB105	GFP-Cas9-NLS/guides pneo-150 Pandora neocal
vHB106	GFP-Cas9-NLS/guides pneo-289 Pandora neocal
vHB107	GFP-Cas9-NLS/guides pneo-177 Pandora neocal
vHB108	GFP-Cas9-NLS/guides pneo-801 Pandora neocal
vHB113	GFP-Cas9-NLS/guides pneo-77 Pandora neocal
vHB114	GFP-Cas9-NLS/guides pneo-153 Pandora neocal
vHB115	GFP-Cas9-NLS/guides pneo-228 Pandora neocal
vHB116	GFP-Cas9-NLS/guides pneo-238 Pandora neocal
vHB117	GFP-Cas9-NLS/guides pneo-248 Pandora neocal
vHB118	GFP-Cas9-NLS/guides pneo-251 Pandora neocal
vHB119	GFP-Cas9-NLS/guides pneo-254 Pandora neocal
vHB120	GFP-Cas9-NLS/guides pneo-264 Pandora neocal
vHB121	GFP-Cas9-NLS/guides pneo-301 Pandora neocal
vHB122	GFP-Cas9-NLS/guides pneo-315 Pandora neocal
vHB123	GFP-Cas9-NLS/guides pneo-321 Pandora neocal
vHB124	GFP-Cas9-NLS/guides pneo-325 Pandora neocal
vHB125	GFP-Cas9-NLS/guides pneo-365 Pandora neocal
vHB126	GFP-Cas9-NLS/guides pneo-383 Pandora neocal
vHB127	GFP-Cas9-NLS/guides pneo-385 Pandora neocal
vHB128	GFP-Cas9-NLS/guides pneo-417 Pandora neocal
vHB129	GFP-Cas9-NLS/guides pneo-459 Pandora neocal
vHB136	GFP-Cas9-NLS/guides pneo-187 Pandora neocal
vHB137	GFP-Cas9-NLS/guides pneo-198 Pandora neocal
vHB138	GFP-Cas9-NLS/guides pneo-221 Pandora neocal
vHB139	GFP-Cas9-NLS/guides pneo-231 Pandora neocal
vHB140	GFP-Cas9-NLS/guides pneo-250 Pandora neocal
vHB141	GFP-Cas9-NLS/guides pneo-261 Pandora neocal
vHB142	GFP-Cas9-NLS/guides pneo-276 Pandora neocal
vHB143	GFP-Cas9-NLS/guides pneo-304 Pandora neocal
vHB144	GFP-Cas9-NLS/guides pneo-313 Pandora neocal
vHB145	GFP-Cas9-NLS/guides pneo-322 Pandora neocal
vHB146	GFP-Cas9-NLS/guides pneo-342 Pandora neocal
vHB147	GFP-Cas9-NLS/guides pneo-344 Pandora neocal
vHB148	GFP-Cas9-NLS/guides pneo-407 Pandora neocal
vHB149	GFP-Cas9-NLS/guides pneo-414 Pandora neocal
vHB150	GFP-Cas9-NLS/guides pneo-425 Pandora neocal
vHB151	GFP-Cas9-NLS/guides pneo-429 Pandora neocal

vHB152	GFP-Cas9-NLS/guides pneo-443 Pandora neocal	
vHB153	GFP-Cas9-NLS/guides pneo-544 Pandora neocal	
vHB154	GFP-Cas9-NLS/guides mk-236	
vHB155	cis-complementation MCP	
vc241	mRFP	193453
vHB178	vHB66 + 3xHA	193457
vHB179	vHB66 + 3xFLAG	193458

Source supplementary.

Fig S1b

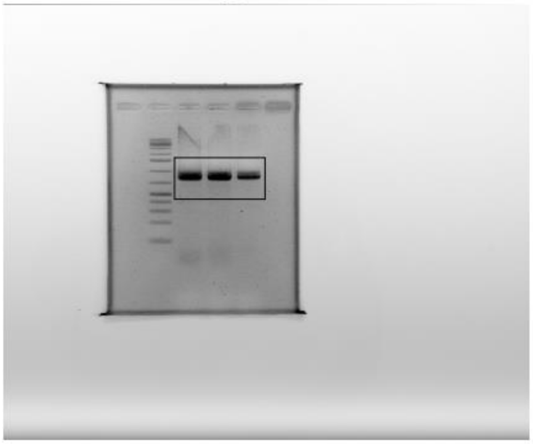

Fig S2c

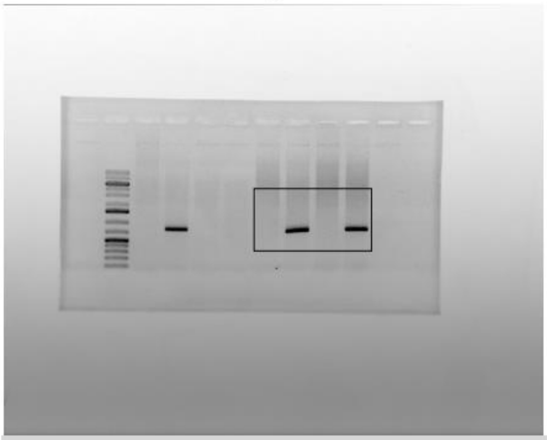

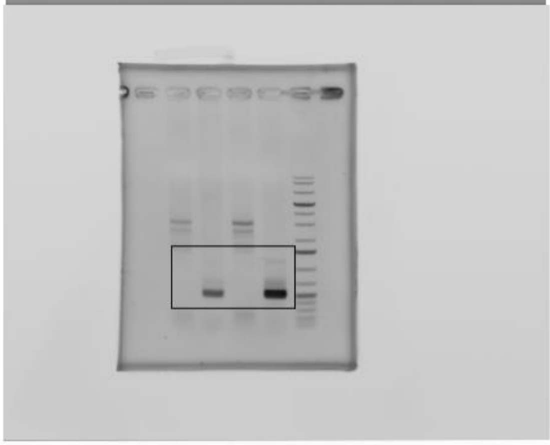

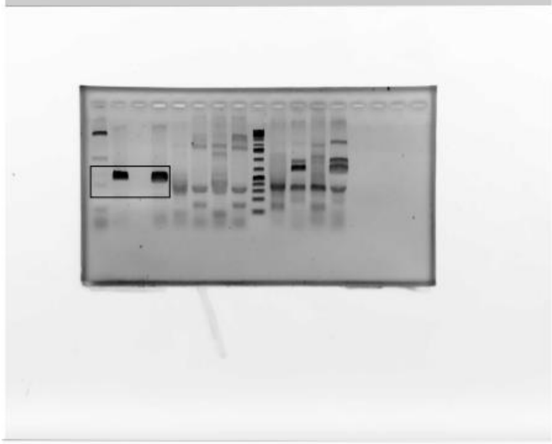

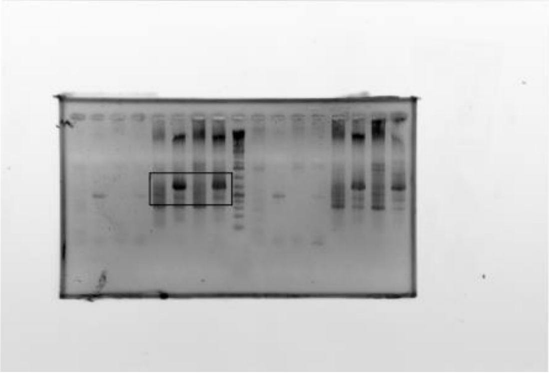

Fig S2d

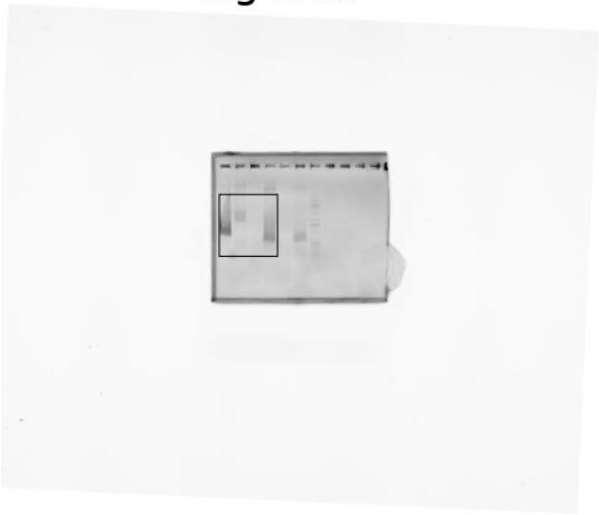

Fig S3a

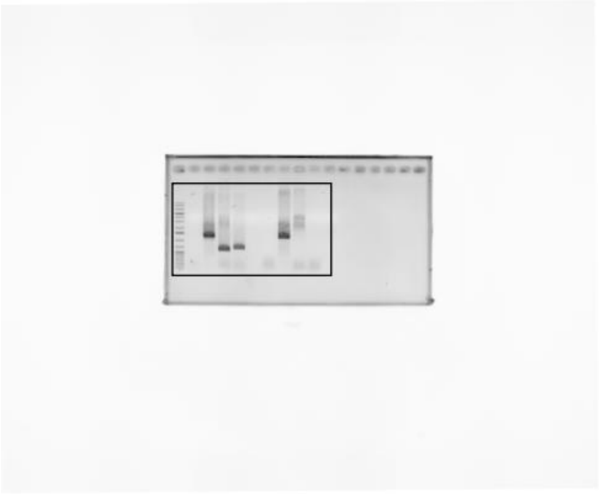

Fig S3a (cont)

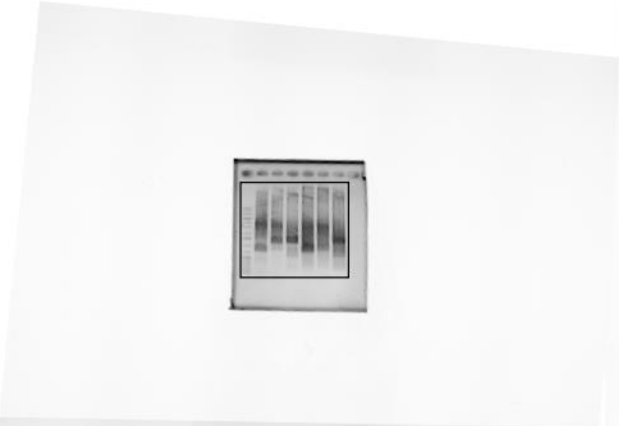

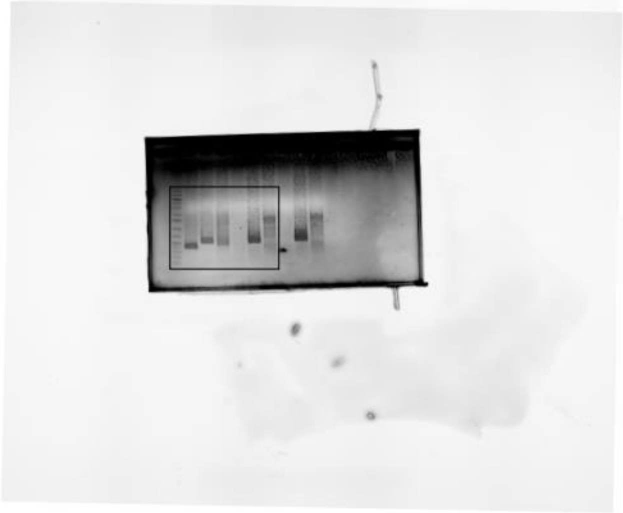

Fig S4b

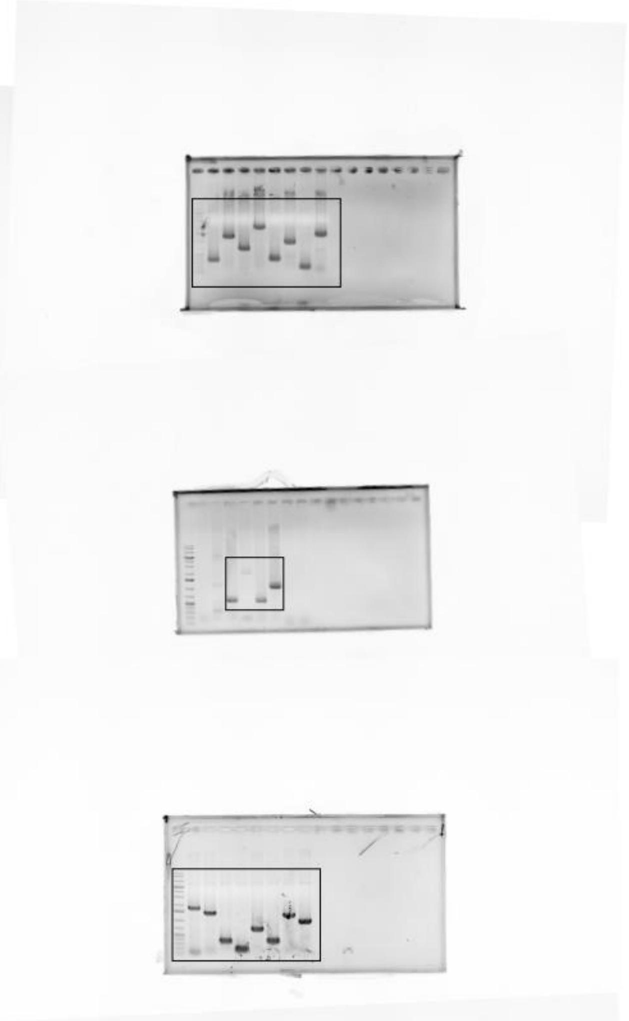

Fig S4b (cont)

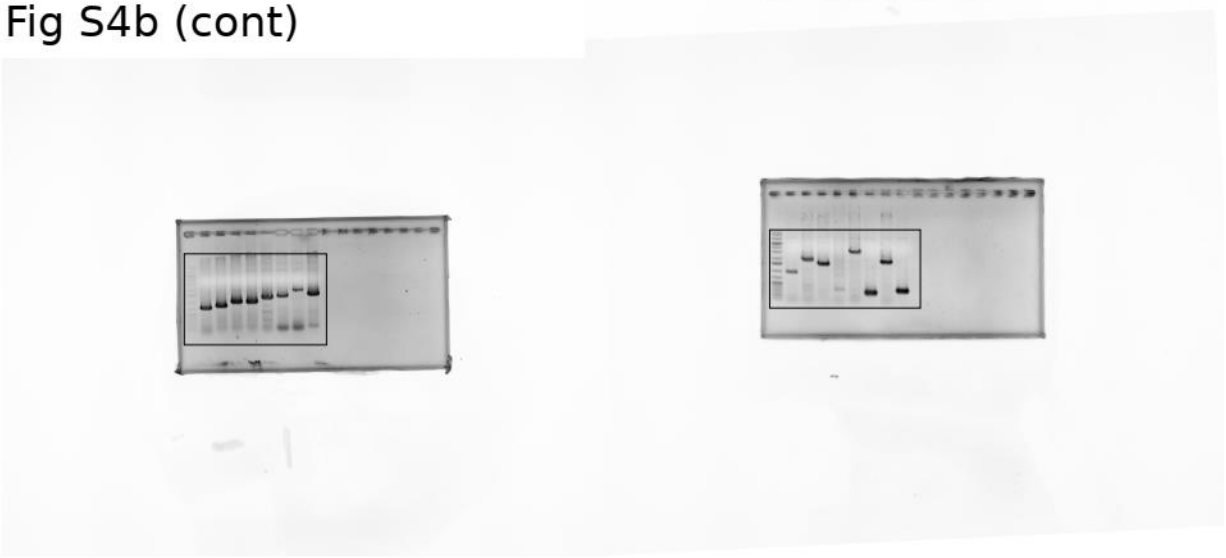

Fig S4b (cont)

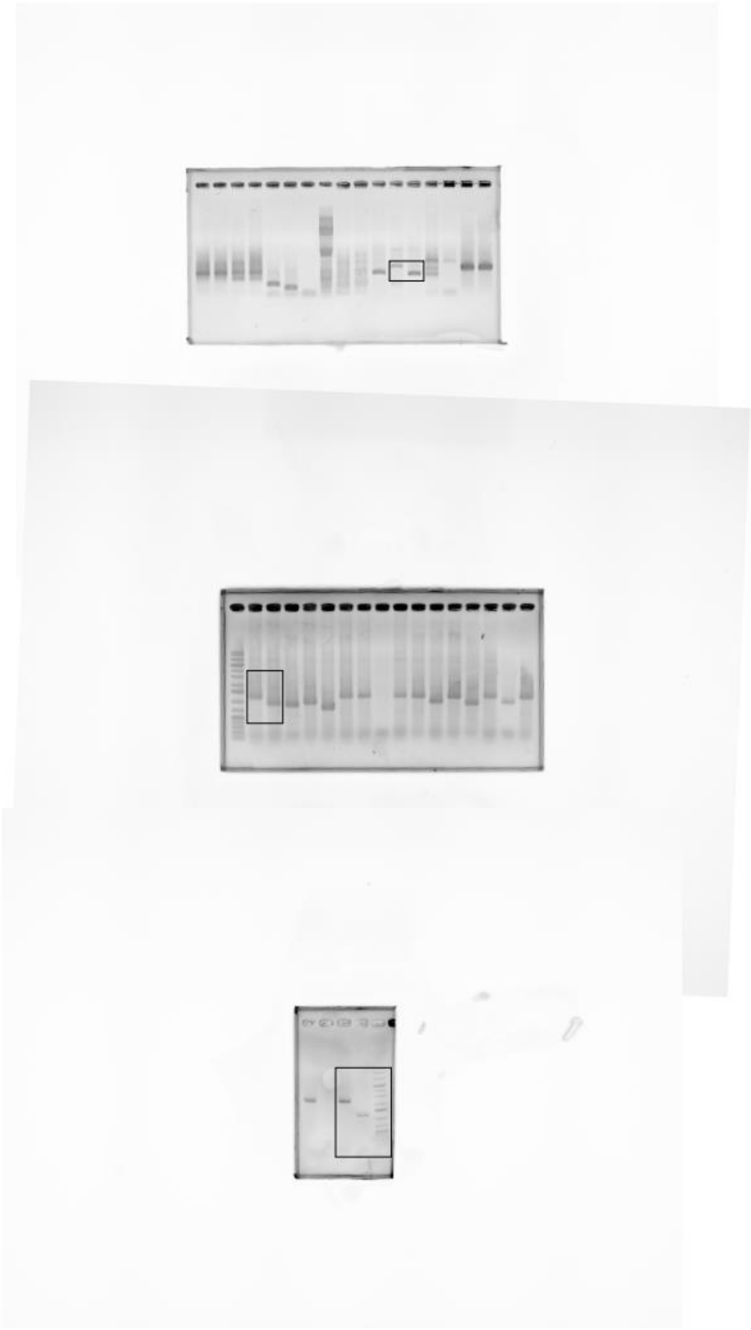

Fig S6b

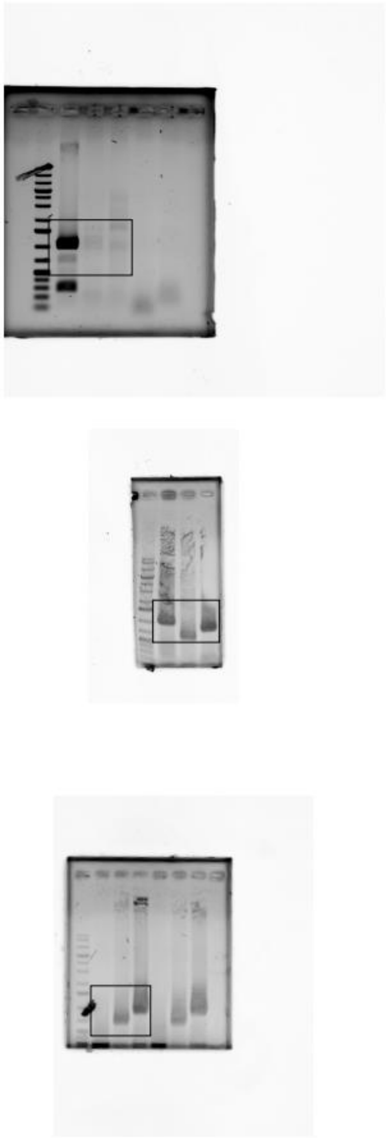

Supplement: Supplementary file 1 — Supplementary Information [file 41467_2023_36145_MOESM1_ESM.pdf]
