# Supplementary material for: Evolution of giant pandoravirus revealed by CRISPR/Cas9
Source: Nat Commun. 2023 Jan 26;14:428. doi: 10.1038/s41467-023-36145-4 (PMC9879987; doi:10.1038/s41467-023-36145-4)
Supplement: Supplementary file 4 — Reporting Summary [file 41467_2023_36145_MOESM4_ESM.pdf]

nature portfolio

Corresponding author(s): Hugo Bisio. Chantal Abergel

Last updated by author(s): Nov 15, 2022

Reporting Summary

Nature Portfolio wishes to improve the reproducibility of the work that we publish. This form provides structure for consistency and transparency in reporting. For further information on Nature Portfolio policies, see our Editorial Policies and the Editorial Policy Checklist.

Statistics

For all statistical analyses, confirm that the following items are present in the figure legend, table legend, main text, or Methods section.

n/a	Confirmed	
☐	☒	The exact sample size (*n*) for each experimental group/condition, given as a discrete number and unit of measurement
☐	☒	A statement on whether measurements were taken from distinct samples or whether the same sample was measured repeatedly
☐	☒	The statistical test(s) used AND whether they are one- or two-sided<br>*Only common tests should be described solely by name; describe more complex techniques in the Methods section.*
☒	☐	A description of all covariates tested
☒	☐	A description of any assumptions or corrections, such as tests of normality and adjustment for multiple comparisons
☐	☒	A full description of the statistical parameters including central tendency (e.g. means) or other basic estimates (e.g. regression coefficient) AND variation (e.g. standard deviation) or associated estimates of uncertainty (e.g. confidence intervals)
☐	☒	For null hypothesis testing, the test statistic (e.g. *F*, *t*, *r*) with confidence intervals, effect sizes, degrees of freedom and *P* value noted<br>*Give P values as exact values whenever suitable.*
☒	☐	For Bayesian analysis, information on the choice of priors and Markov chain Monte Carlo settings
☒	☐	For hierarchical and complex designs, identification of the appropriate level for tests and full reporting of outcomes
☒	☐	Estimates of effect sizes (e.g. Cohen's *d*, Pearson's *r*), indicating how they were calculated

Our web collection on statistics for biologists contains articles on many of the points above.

Software and code

Policy information about availability of computer code

Data collection	AxioVision Rel (4.8). Evolution CapT-Edge.
Data analysis	ApE, A plasmid editor, version 2.0.49.0. Graphpad Prism 8. PyMOL (Anaconda3). Clustal omega (version 1.2.4). ImageJ. OrthoFinder (version 2.5.4).

For manuscripts utilizing custom algorithms or software that are central to the research but not yet described in published literature, software must be made available to editors and reviewers. We strongly encourage code deposition in a community repository (e.g. GitHub). See the Nature Portfolio guidelines for submitting code & software for further information.

Data

Policy information about availability of data

All manuscripts must include a data availability statement. This statement should provide the following information, where applicable:
- Accession codes, unique identifiers, or web links for publicly available datasets
- A description of any restrictions on data availability
- For clinical datasets or third party data, please ensure that the statement adheres to our policy

The authors declare that the data supporting the findings of this study are available within the paper and its supplementary information files. Source data are provided with this paper. Plasmids generated in this study can be obtenaid from Addgene or upon request to the authors. Viral strains can be obtained upon request to the authors. viral genomes: Pandoravirus kuranda (ON887157)

Human research participants

Policy information about studies involving human research participants and Sex and Gender in Research.

Reporting on sex and gender	N/A
Population characteristics	N/A
Recruitment	N/A
Ethics oversight	N/A

Note that full information on the approval of the study protocol must also be provided in the manuscript.

Field-specific reporting

Please select the one below that is the best fit for your research. If you are not sure, read the appropriate sections before making your selection.

☒ Life sciences ☐ Behavioural & social sciences ☐ Ecological, evolutionary & environmental sciences

For a reference copy of the document with all sections, see nature.com/documents/nr-reporting-summary-flat.pdf

Life sciences study design

All studies must disclose on these points even when the disclosure is negative.

Sample size	No minimum sample size was calculated. Experiments were performed with viral productions of 10^9, two orders of magnitude higher than the limit of detection for viral particle counting. Similar sample size are commonly reported in the literature.
Data exclusions	No data was excluded
Replication	All experiments were performed in biological triplicates. All attempts at replication were successful.
Randomization	All experiments were allocated randomly accompanied by appropriate positive and negative controls.
Blinding	Investigators were blinded to ID during quantifications

Reporting for specific materials, systems and methods

We require information from authors about some types of materials, experimental systems and methods used in many studies. Here, indicate whether each material, system or method listed is relevant to your study. If you are not sure if a list item applies to your research, read the appropriate section before selecting a response.

Materials & experimental systems

n/a	Involved in the study	
☐	☒	Antibodies
☐	☒	Eukaryotic cell lines
☒	☐	Palaeontology and archaeology
☒	☐	Animals and other organisms
☒	☐	Clinical data
☒	☐	Dual use research of concern

Methods

n/a	Involved in the study	
☒	☐	ChIP-seq
☒	☐	Flow cytometry
☒	☐	MRI-based neuroimaging

Antibodies

Antibodies used	anti FLAG tag (Catalog # MA1-91878) Thermo Fisher LOT: XF348839A (1/1000 dilution).
Validation	https://www.thermofisher.com/antibody/product/MA1-91878-HRP.html?gclid=EAIaIQobChMI9JL71uaw_AIVlJBoCR2GzQhbEAAYASAAEgLWTvD_BwE&ef_id=EAIaIQobChMI9JL71uaw_AIVlJBoCR2GzQhbEAAYASAAEgLWTvD_BwE:G:s&s_kwcid=AL!3652!3!459737518508!!!g!!!10950825775!106531320406&cid=bid_pca_aup_r01_co_cp1359_pjt0000_bid00000_0se_gaw_dy_pur_con

Eukaryotic cell lines

Policy information about cell lines and Sex and Gender in Research

Cell line source(s)	Acanthamoeba castellanii (Douglas) Neff (American Type Culture Collection 30010TM)
Authentication	N/A
Mycoplasma contamination	N/A
Commonly misidentified lines (See ICLAC register)	N/A
